# The mitochondrial aspartate transporter Ucp4a regulates muscle aging and animal lifespan in *Drosophila melanogaster*

**DOI:** 10.1371/journal.pone.0323980

**Published:** 2025-08-14

**Authors:** Minwoo Baek, Wijeong Jang, Changsoo Kim

**Affiliations:** School of Biological Sciences and Technology, Chonnam National University, Gwangju, South Korea; BSRC Alexander Fleming: Biomedical Sciences Research Center Alexander Fleming, GREECE

## Abstract

Mild distress of mitochondria extends animal lifespan, yet the underlying mechanisms are not completely understood. Here we screened mitochondrial proteins for effects on longevity and found that flies mutant in Uncoupling protein 4a (*Ucp4a*), which encodes a mitochondrial aspartate transporter, have extended lifespans. Tissue-specific experiments revealed knockdown of *Ucp4a* in muscles, but not neurons, fat, or intestine, to extend lifespan and also eliminate polyubiquitinated protein aggregates, which accumulate with aging and are associated with lifespan. These findings suggest a retrograde mitochondrial signaling process initiated by reduced cytosol aspartate level culminates in muscle protein aggregate removal and lifespan extension.

## Introduction

Mitochondria are subcellular organelles that utilize an electron transport chain (ETC) to produce cellular energy and also synthesize numerous metabolites that efflux to the cytosol [1–3]. Mild knockdown of mitochondrial ETC proteins prolongs lifespan, a phenomenon that has been observed in diverse organisms including *C. elegans*, *Drosophila,* and mice [4–7]. In mammalian cells, ETC perturbation or mitochondrial distress represses the mechanistic target of rapamycin complex 1 (mTORC1) pathway through activation of the transcription factor ATF4 [2,8–12]. Studies in *Drosophila* muscle have found ETC perturbation to repress systemic insulin signaling through expression of ImpL2, an inhibitor of *Drosophila* insulin-like peptides (Dilps) [13,14]. Repression of either mTORC1 or insulin signaling is established to extend lifespan [15–19]; however, the mechanisms underlying this mitochondrial-distress-mediated life extension are not yet completely understood.

Aspartate (asp), a proteogenic amino acid, is synthesized in the mitochondrial matrix from glutamate and oxaloacetic acid (OAA), a tricarboxylic acid (TCA) cycle metabolite [20]. Of note, asp synthesis requires integral mitochondrial function. In mammalian cells, treatment with an ETC inhibitor depletes asp due to impairment of NADH flux, which is required for integrity of the TCA cycle [12,21]. Perturbation of asp synthesis impairs cell proliferation, and in endothelial cells impairs the cytosolic mTORC1 pathway [12,21–23]. Here we show that Uncoupling protein 4 a (Ucp4a), a mitochondrial asp transporter [24], regulates muscle aging and lifespan in *Drosophila*.

## Materials and methods

### 2.1. Fly husbandry

*Ucp4a*^*G1388*^, *da*-, *elav*-, *MHC*-, *mef*-, *cad*- and *C574*-*Gal4* lines were from the *Bloomington Stock Center*. *UAS*-*Ucp4a RNAi (#6162* and *#102571*) were provided by the *VDRC Stock Center*. Fly culture and crosses were performed on standard fly food containing yeast, cornmeal, and sugar, and the flies were raised at 25 °C. The *w*^*1118*^ line was used as a wild-type genetic background.

### 2.2. Lifespan assay

All flies, including *Ucp4a*^*G1388*^, *Gal4* lines and *UAS-ucp4a RNAi #6162, #102571*, were backcrossed to the *w*^*1118*^ line for six generations to minimize genomic background differences. Flies (1 ~ 2 days old) were collected by anesthetization with CO_2_ gas. All flies were kept in a humidified (50%), temperature-controlled incubator with a 12 h on/off light cycle at 25°C. Flies (20 per vial) were transferred to fresh food every three days and scored for death. Significant differences in lifespan were assessed using the log-rank test method in *GraphPad Prism 5*.

### 2.3. Capillary feeding (CAFE) assay

Quantification of food uptake was performed by capillary feeding assay as described previously [25] with slight modification. Briefly, five flies were placed in vials with wet glass paper for a water source and a capillary food source (5% sucrose, 5% yeast extract). Feeding was monitored every six hours, at which time fresh capillaries were placed and the feeding amount calculated.

### 2.4. Quantitative RT-PCR

Total RNA was extracted with TRIzol reagent (Invitrogen) following manufacturer protocols. Samples were treated with DNase, and the RNA concentrations measured and equalized. Complementary DNA was synthesized using a cDNA synthesis kit (Bioneer). Quantitative RT-PCR was performed using QuantiTech SYBR Green (Qiagen), and DNA amount was monitored with a Rotor-Gene Q (Qiagen). Equalized amplicons of *Rp49* were used to normalize. Primer sequences were as follows: *Ucp4a*, 5’-TTCGCCTGCACTTACATCGT-3’and 5’-CACCGCTATAGACGACGTGT-3’.

### 2.5. Immunostaining

Total polyubiquitinylated protein and the sarcomere structure of the indirect flight muscle were evaluated as previously described [26]. Briefly, adult flies were fixed with 4% paraformaldehyde in PBS for 1 h, embedded in OCT compound (Fisher Scientific), and frozen with liquid nitrogen. Samples were then processed by a cryomicrotome (Leica). After post-fixing with 4% paraformaldehyde in PBS, samples were permeabilized with 0.2% Triton X-100 buffer in PBS and blocked with 5% BSA solution in PBS for 1 h. Afterwards, the samples were incubated with anti-ubiquitinylated proteins antibody (Millipore, 1:200) and phalloidin AlexaFluor-488 (Invitrogen 1:1000) overnight at 4 °C. Anti-mouse IgG Alexa Flore 568 (Invitrogen) was used as the secondary antibody (1:1000). Samples were mounted with Prolong Gold Antifade Reagent with DAPI (Invitrogen) and imaged with a Leica confocal microscope (Leica); finally, the images were analyzed by the ImageJ software.

### 2.6. Locomotive assay

Flies (1 ~ 3 days old) were collected by anesthetization with CO_2_ gas. Twenty male flies per vial, which were kept in a humidified (50%) incubator at 25°C, were transferred to fresh food every three days. For locomotive assays, flies (5, 10, 30, 45, 50, 60 day old) were transferred to an empty vial directly from the food vial, idled for 10 min, and then tapped to the bottom. The number of flies that climbed to reach a 7 cm cutline within 10 sec was counted. Data are shown as mean±SEM and were analyzed using *GraphPad Prism 6.0*.

### 2.7. Data analysis

The statistical significance of differences in standard error of the mean (SEM) or standard deviation (SD) of the mean values was obtained using two-way ANOVA with Sidak’s multiple comparisons test. Kaplan-Meier survival curves was used for lifespan and survival analysis with log-rank tests. Prism software (*GraphPad* version 6.0, San Diego, USA) was used to calculate *p*-values. Results were considered statistically significant at levels *p < 0.05, **p < 0.01, ***p < 0.001, ****p < 0.0001.

## Results

**Fig 1:**
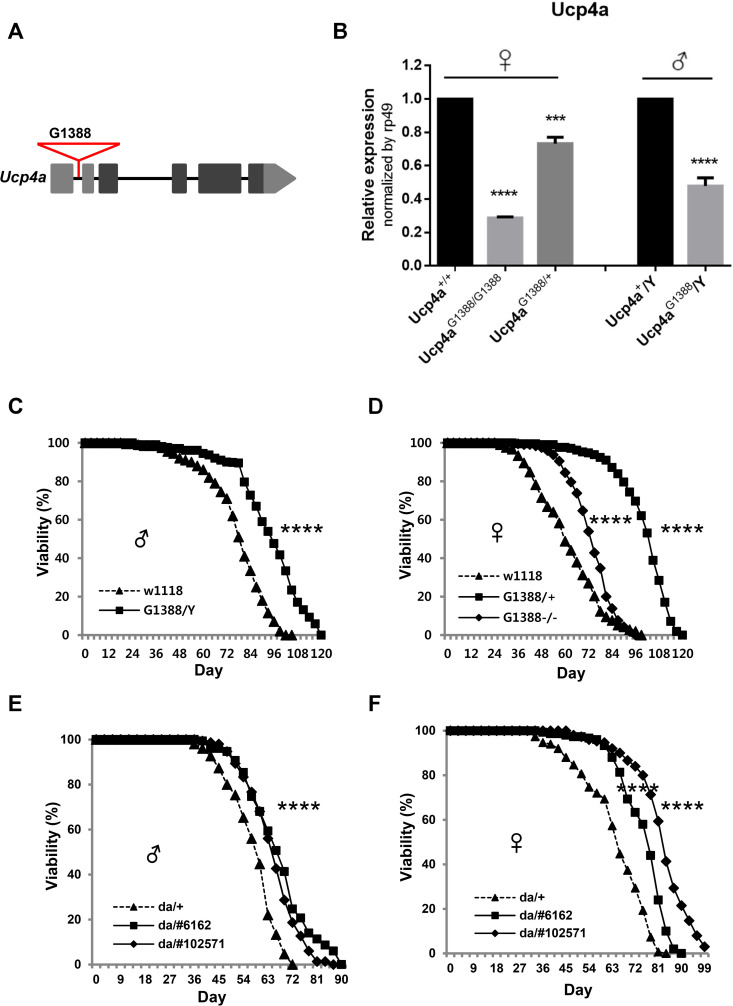
Prolongation of lifespan by mutation and knockdown of *Ucp4a.* (A) The *Ucp4a* mutant line. G1388 is an EP element inserted into the first intron of the *Ucp4a* gene. Gray and black boxes denote noncoding and coding sequences, respectively. (B) qRT-PCR of *Ucp4a* normalized to *rp49*. Error bars indicate ±SEM of three independent experiments. Student’s *t*-test, ***, *p* < 0.001. ****, *p* < 0.0001. (C,D) Viability of *Ucp4a*^*G1388*^ hemizygous mutant (*ucp4a*^*G1388*^/Y) and *w*^*1118*^ (genetic control for EP line) male flies (C), and *Ucp4a*^*G1388*^ heterozygous mutant (*ucp4a*^*G1388*^/^+^), homozygous mutant (*ucp4a*^*G1388*^/ *ucp4a*^*G1388*^) and *w*^*1118*^ female flies (D). W1118 denotes *W*^*1118*^/Y. G1388/Y denotes *Ucp4a*^*G1388*^/Y. G1388/+ denotes *Ucp4a*^*G1388*^/^+^. G1388-/- denotes *Ucp4a*^*G1388*^/*Ucp4a*^*G1388*^. n = 400 for each curve. ****, *p* < 0.0001, log rank test. (E,F) Viability of *Ucp4a* whole-body knockdown male (E) and female (F) flies. da/#6162 and da/#102571 indicate *da-Gal4* > *UAS-Ucp4a RNAi* #*6162* and #*102571*, respectively. da/+ indicates *da*-*Gal4*/+ , used as control. n = 400 for each curve. ****, *p* < 0.0001, log rank test.

We carried out a screening that identified a longevity line with an EP-element [27] inserted into the first intron of *Ucp4a* (hereafter we term the *Ucp4a* mutant line as *Ucp4a*^*G1388*^) (Fig 1A). qRT-PCR showed that *Ucp4a* mRNA was reduced in *Ucp4a*^*G1388*^ hemizygous males (*Ucp4a*^*G1388*^/Y), and heterozygous (*Ucp4a*^*G1388*^/^+^) and homozygous females (*Ucp4a*^*G1388*^/*Ucp4a*^*G1388*^ or *Ucp4a* ^-^/^-^) (Fig 1B). *ucp4a* hemizygous males (*Ucp4a*^*G1388*^/Y) exhibited longer lifespan, with an average 18.5% extension (Fig 1C), and *ucp4a* homozygous females (*Ucp4a*^*G1388*^/*Ucp4a*^*G1388*^ or *Ucp4a* ^-^/^-^) exhibited a comparable 19.0% increase (Fig 1D). Notably, *ucp4a* heterozygous females (*Ucp4a*^*G1388*^/^*+*^ or *Ucp4a* ^+^/^-^) exhibited a dramatic lifespan increase of 66.7% (Fig 1D).

**Fig 2:**
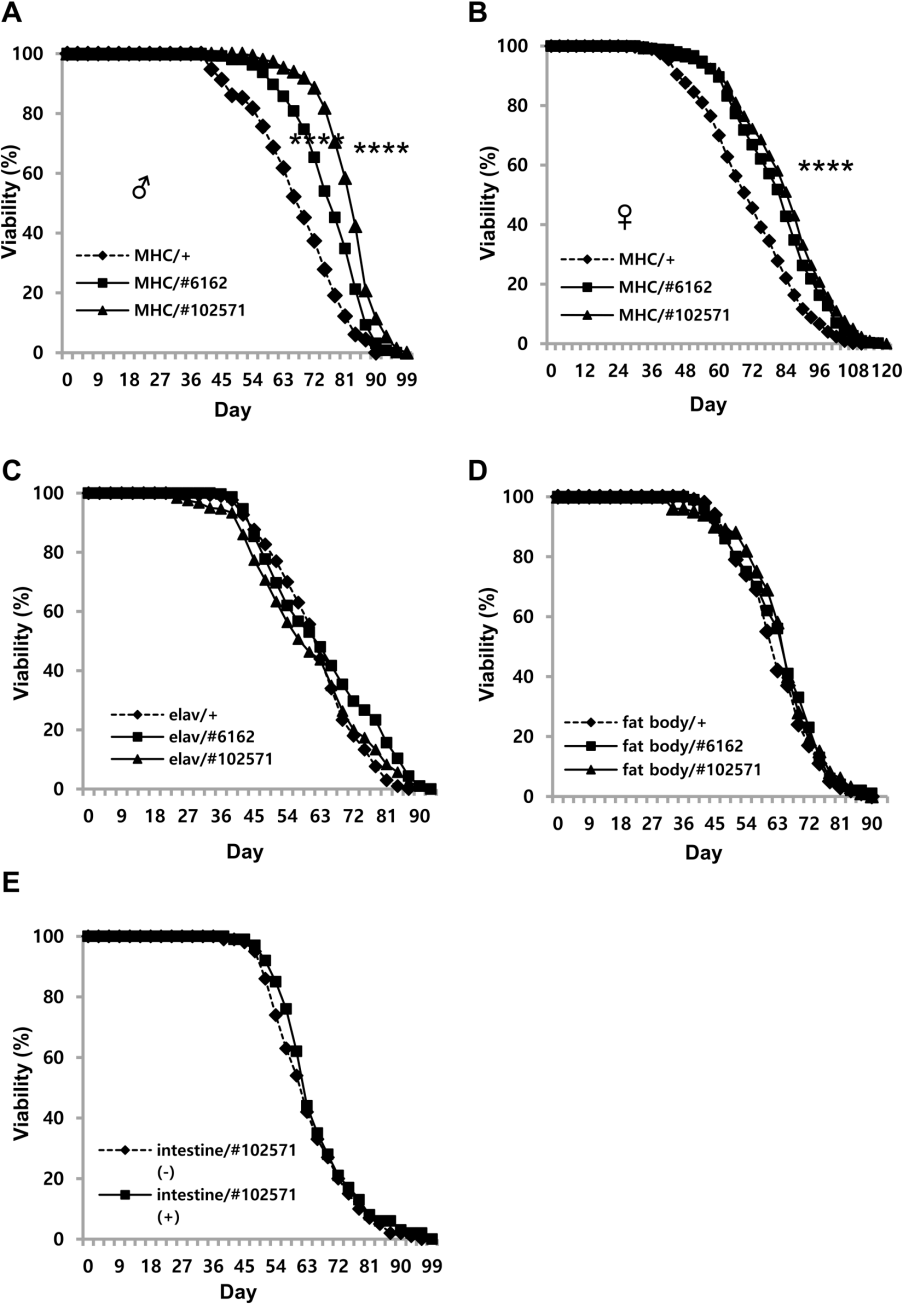
Prolongation of lifespan by tissue-specific knockdown of *Ucp4a.* (A,B) Viability of *Ucp4a* muscle-specific knockdown male (A) and female (B) flies. MHC/+ denotes *MHC-Gal4*/+ . MHC/#6162 denotes *MHC-Gal4*/+ ; *Ucp4a RNAi* #*6162*. MHC/#102571 denotes *MHC-Gal4*/+ ; *Ucp4a RNAi* #*102571*. n = 400 for each curve. ****, *p* < 0.001, log rank test. (C) Viability of *Ucp4a* knockdown male by neuronal Gal4 (*elav-Gal4*). Elav/+ denotes *elav-Gal4*/+ . Elav/#6162 denotes *elav-Gal4*/+ ; *ucp4a RNAi* #6162. Elav/#102571 denotes *elav-Gal4*/+ ; *ucp4a RNAi #102571*. (D) Viability of *Ucp4a* knockdown male by fat body Gal4 (*cad-Gal4*). Fat body/+ denotes *cad-Gal4*/+ . Fat body/#6162 denotes *cad-Gal4*/+ ; *ucp4a RNAi* #*6162*. Fat body/#102571 denotes *cad-Gal4*/+ ; *ucp4a RNAi* #*102571*. (E) Viability of *Ucp4a* knockdown male by drug-inducible intestinal Gal4 (*c574-Gal4*). Intestine/#102571 denotes *c574-Gal4*/+ ; *ucp4a RNAi* #*102571*. (-), (+) denote administration of drug (RU486, 50 µg/ml) in food.

Knocking down of *Ucp4a* in whole cells with *Ucp4a* RNAi driven by *da-Gal4*, a ubiquitous *Gal4* driver, in two distinct RNAi lines (*da-Gal4* > *UAS-Ucp4a RNAi #6162, #102571*) extended lifespan in males and females by 20% and 30%, respectively (Fig 1E,F). Subsequently, *elav-*, *MHC-*, *cad-*, and *c574*-*Gal4* tissue-specific drivers were used to drive *Ucp4a* RNAi in neurons, muscle, fat body, and intestine, respectively. Interestingly, the muscle-specific *Gal4* driver was effective in extending male lifespan by 21.7% (Fig 2A), comparable to the ubiquitous driver (Fig 1E,F). Likewise, in females, the muscle *Gal4* driver extended lifespan by 26%, similar to the 31% of the whole-body driver (Fig 2B). Other *Gal4* drivers did not increase longevity (Fig 2C–E). *UAS-Ucp4a #6162* was slightly less effective than *Ucp4a RNAi #102571* (Figs 1F,2A). Taken together, these data suggest that muscle is the source of the *Ucp4a*-mediated effect on lifespan.

**Fig 3:**
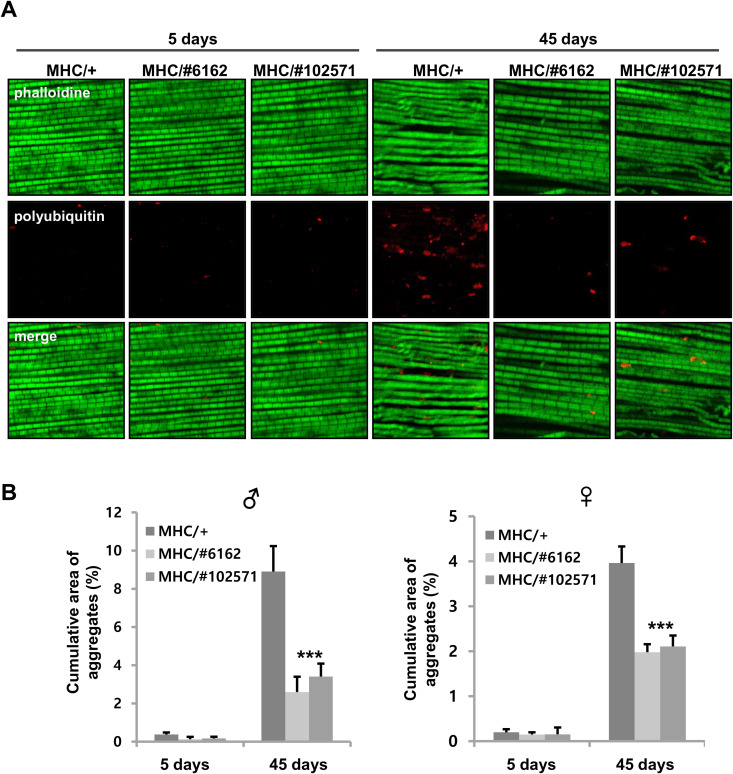
Polyubiquitinated protein aggregates in muscle with age. (A) Confocal images of adult male thorax (indirect flight muscle) sections co-stained with polyubiquitinated protein antibody (red) and phalloidin (green). MHC/+ denotes *MHC-Gal4*/+ . MHC/#6162, 102571 indicates *MHC-Gal4* > *UAS-Ucp4a RNAi* #*6162*, #*102571*, respectively. (B) Cumulative area of aggregates normalized to total visualized area from Fig 3A. Error bars indicate SD for n = 10 muscles. ***, *p* < 0.001, two-way ANOVA with Sidak’s multiple comparisons test.

**Fig 4:**
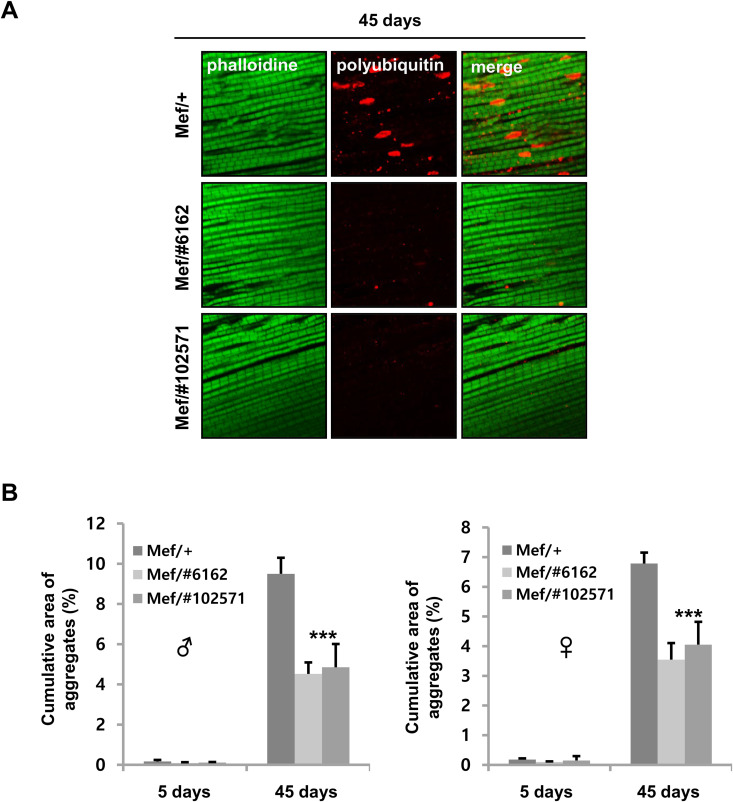
Polyubiquitinated protein aggregates in muscle with age. (A) Immunohistochemical staining of adult thorax (indirect flight muscle) sections co-stained with polyubiquitinated protein antibody (red) and phalloidin (green). 45-day-old male flies. Mef/+ (control) denotes *Mef-Gal4/*+ . Mef/#6162, #102571 indicates *Mef*-*Gal4* > *UAS-Ucp4a RNAi* #*6162*, #*102571*. (B) Cumulative area of aggregates normalized to total visualized area. Averages and SD of the mean were derived for n = 10 muscles. ***, *p* < 0.001, two-way ANOVA with Sidak’s multiple comparisons test.

An association of muscle aging with lifespan has previously been demonstrated [13,28,29], prompting us to investigate whether *Ucp4a* reduction in muscle delays muscle aging. Accumulation of polyubiquitinated proteins is a hallmark of muscle aging [29]. We confirmed polyubiquitinated aggregates to be increased in normal aged muscles (Fig 3). Meanwhile, the *ucp4a* mutant (S1 Fig) and muscle-specific *Ucp4a* knockdown lines (*MHC-Gal4* and *mef-Gal4*) exhibited reduced polyubiquitinated aggregates in aged muscles (Figs 3,4).

**Fig 5:**
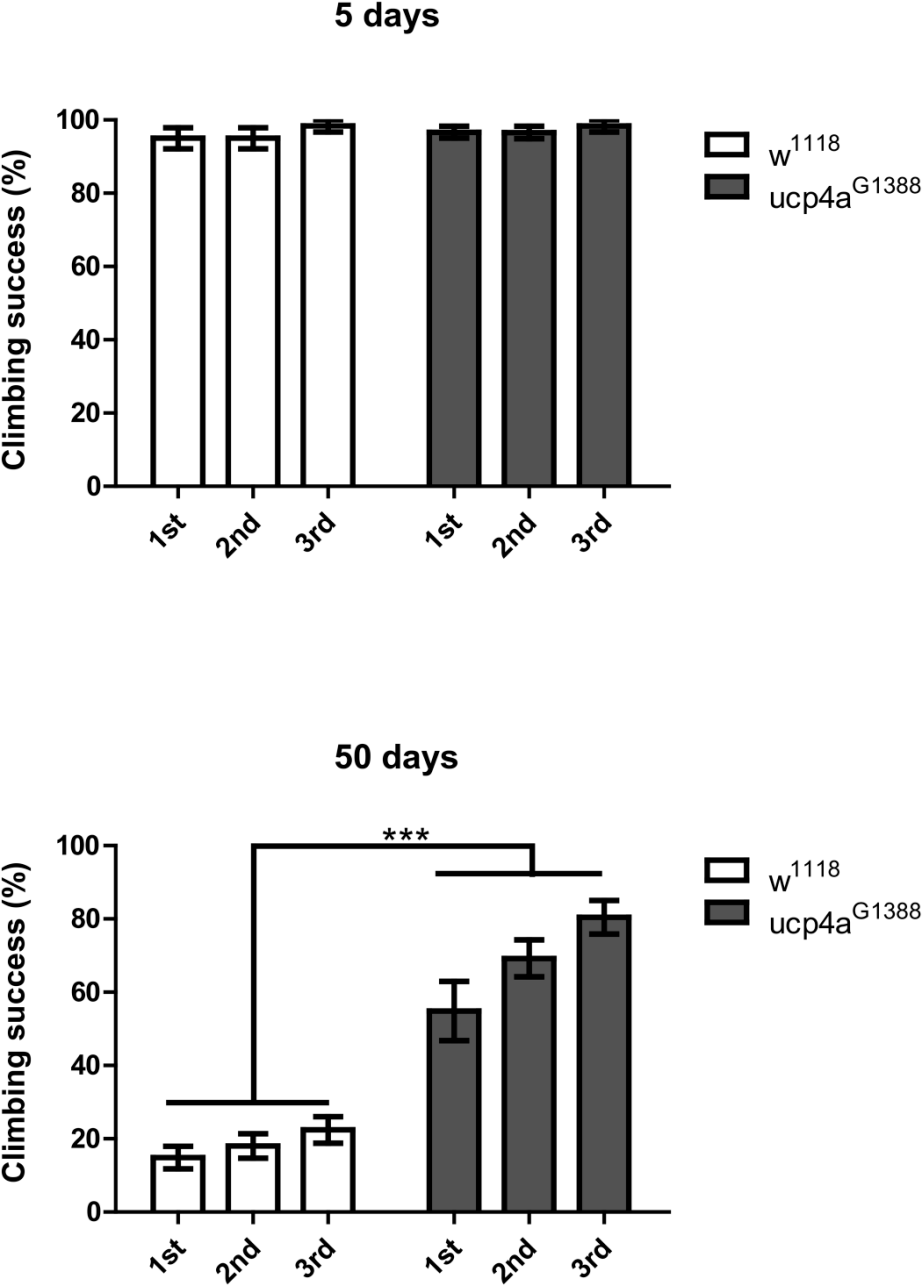
Climbing assay. The number of male flies per vial that climbed 7 cm within 10 sec after tapping was counted. *Ucp4a*^*G1388*^ indicates *Ucp4a* hemizygous mutant flies (*Ucp4a*^*G1388*^/Y). 1^st^, 2^nd^, 3^rd^ denote individual trials. Error bars indicate ±SEM. n = 100. ***, *p* < 0.001, Student’s *t*-*t*est.

Longevity phenotypes have also been linked to food consumption [15,29]; accordingly, we explored this possibility in our flies, determining food uptake using the CAFÉ assay [25]. The results showed no reduction of food consumption in the *ucp4a* mutant (S2 Fig). We further tested whether reduction of protein aggregates leads to healthy muscles using climbing assays. In these assays, the *ucp4a* mutant exhibited better climbing ability, suggesting that *ucp4a* mutant flies possess healthier muscles (Fig 5). Flies with *Ucp4a* knockdown in muscle also exhibited better climbing ability (S3 Fig).

## Discussion

Here we show that mutation in the *Ucp4a* gene can extend lifespan without restricting feeding. Remarkably, the life-extension effect of *Ucp4a* mutation is specifically due to knockdown of *Ucp4a* in muscles; knockdown in other tissues was not effective in life-extension. We find that protein aggregates, a characteristic of muscle aging, are reduced by *Ucp4a* knockdown in muscles. Consistently, *Ucp4a* mutants and lines with *Ucp4a* knockdown in muscle maintain healthier muscle than control flies, as suggested by observation of enhanced locomotor activity in aged flies.

Our data show that *Ucp4a* hemizygous male and *Ucp4a* homozygous female flies still possess 20 ~ 40% the level of control *Ucp4a* transcripts, suggesting that they still possess some *Ucp4a* activity. Intriguingly, *Ucp4a* heterozygous females, which harbor one wild-type *Ucp4a* allele, exhibited longer lifespans compared to homozygous mutant females, suggesting that milder *Ucp4a* distress is more effective in lifespan extension. This finding is in line with previous reports that mild distress of mitochondria increases lifespan whereas severe distress shortens it [4]. In future, the effect of dose-dependency on lifespan could be tested using *Ucp4a* null mutants if available. Our present findings suggest that proper level of *Ucp4a* activity disturbance in the mitochondria might be a key determinant of lifespan.

Aspartate (Asp) is converted to asparagine (Asn) by the asparagine synthetase (ASNS) enzyme in the cytosol [12], suggesting that *Ucp4a* knockdown is likely to reduce cytosolic Asn. This reduction might be a signal that relates to inhibition of the mTORC1 pathway [12,30–32]. This possibility is supported by a recent finding that inhibition of glutaminolysis, which is required for asp synthesis, activates the ATF4-mediated pathway to suppress mTORC1 activity through expression of the mTORC1 negative regulators Sestrin2 and Redd1 [2,22]. Ultimately, asp reduction-mediated lifespan extension might require inhibition of the mTORC1 pathway. Further confirmatory research remains required.

## Supporting information

S1 Fig Polyubiquitinated protein aggregates in muscle. Confocal images of adult thorax (indirect flight muscle) sections co-stained with polyubiquitinated protein antibody (red) and phalloidin (green, sarcomere structure). 50-day-old male flies. *Ucp4a* denotes *Ucp4a*^*G1388*^/Y.
(TIF)

S2 Fig Quantification of food uptake by capillary feeding assay. Food uptake was measured for five flies (5 ~ 7 day old) over six hours in a vial. Each bar is an average from a triplicated experiment. Two trials are shown. W1118 indicates *W*^*1118*^/Y (male), *W*^*1118*^/ *W*^*1118*^ (female). G1388 indicates *Ucp4a*^*G1388*^/Y(male), *Ucp4a*^*G1388*^/*Ucp4a*^*G1388*^ (female).
(TIF)

S3 Fig Climbing assay. The number of flies per vial that climbed 7 cm within 10 sec after tapping was counted. Male flies of different ages. MHC/+ indicates *MHC-Gal4*/+ . Ucp4a RNAi/+ indicates *UAS-Ucp4a RNAi #6162*/+ . MHC/Ucp4a RNAi indicates *MHC-Gal4*/*UAS-Ucp4a RNAi #6162*. Error bars indicate ±SEM. n = 100. *, *p* < 0.05, Student’s *t*-test.
(TIF)

S1 Data Individual numerical values displayed in all graphs. (XLSX)
