# Supplementary figures and images for: The mitochondrial aspartate transporter Ucp4a regulates muscle aging and animal lifespan in Drosophila melanogaster
Source: PLoS One. 2025 Aug 14;20(8):e0323980. doi: 10.1371/journal.pone.0323980 (PMC12352782; doi:10.1371/journal.pone.0323980)

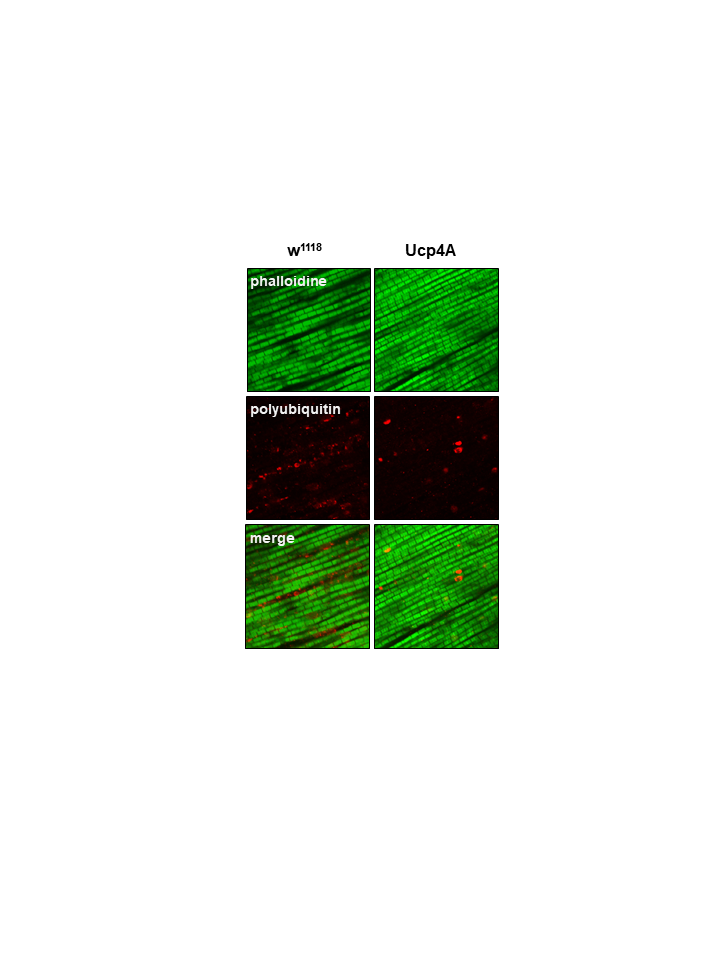

Supplement: S1 Fig — Confocal images of adult thorax (indirect flight muscle) sections co-stained with polyubiquitinated protein antibody (red) and phalloidin (green, sarcomere structure). 50-day-old male flies. Ucp4a denotes Ucp4aG1388/Y. (TIF) [file pone.0323980.s001.tif]

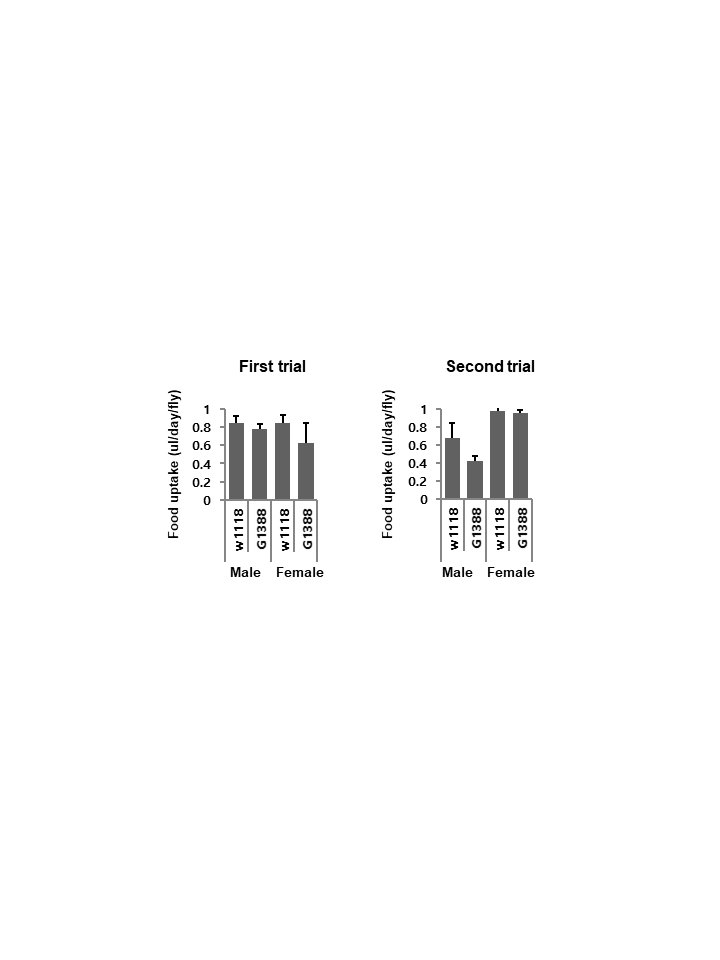

Supplement: S2 Fig — Food uptake was measured for five flies (5 ~ 7 day old) over six hours in a vial. Each bar is an average from a triplicated experiment. Two trials are shown. W1118 indicates W1118/Y (male), W1118/ W1118 (female). G1388 indicates Ucp4aG1388/Y(male), Ucp4aG1388/Ucp4aG1388 (female). (TIF) [file pone.0323980.s002.tif]

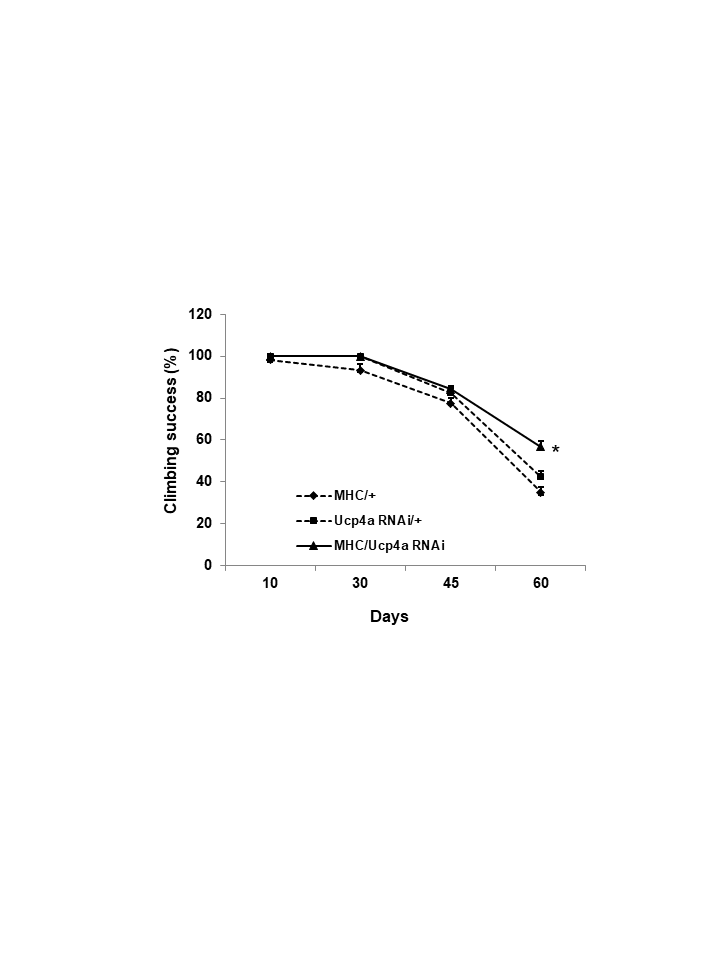

Supplement: S3 Fig — The number of flies per vial that climbed 7 cm within 10 sec after tapping was counted. Male flies of different ages. MHC/+ indicates MHC-Gal4/+ . Ucp4a RNAi/+ indicates UAS-Ucp4a RNAi #6162/+ . MHC/Ucp4a RNAi indicates MHC-Gal4/UAS-Ucp4a RNAi #6162. Error bars indicate ±SEM. n = 100. *, p < 0.05, Student’s t-test. (TIF) [file pone.0323980.s003.tif]
